# The prevalence of altered body image in patients with primary brain tumors: an understudied population

**DOI:** 10.1007/s11060-020-03433-8

**Published:** 2020-02-24

**Authors:** Lindsay Rowe, Elizabeth Vera, Alvina Acquaye, Sonja Crandon, Veeraj Shah, Christine Bryla, Jing Wu, Kathleen Wall, Christine Siegel, Jennifer Reyes, Marta Penas-Prado, Nicole Leggiero, Christine Cordova, Eric Burton, Ramya Antony, Lisa Boris, Orwa Aboud, Yamini Vyas, Peter Mathen, Mark Gilbert, Kevin Camphausen, Tito Mendoza, Terri Armstrong

**Affiliations:** 1 Radiation Oncology Branch, National Cancer Institute, National Institutes of Health, Bethesda, MD USA; 2 Neuro-Oncology Branch, National Cancer Institute, National Institutes of Health, Bethesda, MD USA; 3 University of Maryland, College Park, MD USA; 4 Leidos Biomedical Research, Frederick National Laboratory for Cancer Research Sponsored By the National Cancer Institute, Frederick, MD USA; 5 Department of Symptom Research, Division of Internal Medicine, The University of Texas MD Anderson Cancer Center, Houston, TX USA

**Keywords:** Brain tumors, Quality of life, Body image, Psychological distress

## Abstract

**Purpose:**

Body image (BI) is an important issue for cancer patients, as patients with BI concerns are susceptible to depression, anxiety, difficulty coping, and poor quality of life (QoL). While this concern has been documented in patients with other malignancies, no data exists of this QoL issue in patients with primary brain tumors (PBT).

**Methods:**

A cross-sectional survey of 100 PBT patients was conducted on an IRB approved prospective protocol using structured questionnaires. Participants completed the body image scale (BIS), Appearance Scheme Inventory Revised (ASI-R), MD Anderson Symptom Inventory Brain Tumor (MDASI-BT), and Patient-Reported Outcomes Measurement Information System (PROMIS) Depression, Anxiety, and Psychosocial Impact Positive measures.

**Results:**

The prevalence of clinically significant body image dissatisfaction (BIS ≥ 10) was 28% (95% CI 19–37%), median BIS score was 5 (range 0–27). The median ASI-R composite score was 2.9 (range 1.5–4.7). BIS was significantly correlated with the ASI-R (*r* = 0.53, 95% CI 0.37 to 0.65). The mean PROMIS Depression score was 48.4 (SD = 8.9), PROMIS Anxiety score was 49.4 (SD = 9.9), and PROMIS Psychosocial Illness Impact Positive score was 48.9 (SD = 9.7). BIS was significantly correlated with age, and trended with BMI and sex. The PROMIS Psychosocial Illness Impact Positive and PROMIS Anxiety scores were the most strongly related to BIS.

**Conclusions:**

This study, the first to explore altered body image in PBT patients, revealed clinically significant body image dissatisfaction in nearly 1/3 of patients, similar to other malignancies. These findings underscore the potential contribution of disease and treatment-related body image concerns on psychosocial wellbeing in patients with PBT.

**Electronic supplementary material:**

The online version of this article (10.1007/s11060-020-03433-8) contains supplementary material, which is available to authorized users.

## Introduction

Body image is a multidimensional concept that includes one’s physical appearance and the cognitive, emotional, and relational elements that influence an individual’s sense of identity [1]. It includes objective and subjective factors such as perceptions, feelings, and attitudes toward the body, which can be significantly affected by disease. Since an individual’s body mediates their contact with the outside world, their body image therefore has implications in relationships, sexuality, and self-esteem [2–10].

Body image has been found to be an important concern for cancer patients. It has been noted that the physical signs of disease or its treatment can be a constant reminder of the reality of cancer, leading to adaptation difficulties or the emergence of emotional frailty [2]. Body image has been studied in breast, testicular, prostate, head and neck, melanoma, sarcoma, and gastrointestinal tumors, among others [3–10]. In breast and head and neck cancer body image has been conceptualized to include both disfigurement and dysfunction, and is affected by pre-existing patient characteristics, social factors, environmental factors, and time from treatment [11, 12]. Resultant body image has then been found to affect patients’ social and psychological outcomes, as well as general quality of life. These consequences include higher rates of depression, anxiety, and difficulty coping [13, 14].

While body image concerns and their psychosocial outcomes have been documented in patients with many cancer pathologies, currently no data exists of this quality of life (QoL) issue in patients with primary brain tumors (PBT) [9]. Patients with PBT can suffer from disease related neurologic dysfunction, such as facial asymmetry, hoarse voice, dysarthria, weakness, vision and sensory changes. Treatment-related cosmetic alterations can leave scars, craniofacial deformity, alopecia, steroid related weight gain, and cushingoid appearance. Despite the potential significant impact of these changes on self-esteem, sexuality, and interpersonal relationships, very little is known regarding body image changes in patients with PBT.

The goal of this study was to address the prevalence of body image concerns in PBT patients using validated questionnaires previously used in other cancer populations, and explore any contributing psychological, disease, and treatment related factors. This information is of importance as the prevalence of people in the US living with PBT was estimated to be 688,000 in 2010 and projecting that 86,970 will be diagnosed in 2019 [15, 16].

## Methods

### Patients

Patients enrolled on an Institutional Review Board approved protocol between December 2017 and April 2018 were screened and approached if eligible. This was a cross-sectional design at a single time point. and eligibility requirements were adult (≥ 18 years old) patients with histologically confirmed PBT, with intracranial only disease, who were proficient in English. Informed consent was obtained from all participants. Patient medical data was collected prospectively from patient charts such as age, gender, Karnofsky Performance Score (KPS), height, weight, cancer diagnosis, and previous treatment history.

### Measures

The Body Image Scale (BIS) was used to assess body image dissatisfaction. The questionnaire allows cancer patients to self-rate change in body image after diagnosis or treatment [17]. It is a 10-item questionnaire, with total scores calculated by the sum of responses ranging from 0 to 30. Increasing scores suggest increased body image dissatisfaction. Clinical cutoff for body image dissatisfaction in cancer patients has been suggested as a score of ≥ 10 as used in breast and prostate cancer, and applied in a diverse population of cancer patients with advanced disease [6, 8, 17, 18].

Investment in body image and appearance was assessed by the Appearance Schemas Inventory–Revised, a 20-item scale with scores calculated based on the mean of responses for a Composite score [19]. It consists of two subscales for Self-evaluative and Motivational salience, with higher scores suggesting higher investment in appearance. The scales assess to what extent an individual defines themselves by their physical appearance (self-evaluative) and how much they attend to their appearance (motivational).

Symptom burden was assessed using the MD Anderson Symptom Inventory-Brain Tumor Module (MDASI-BT). The 28-item MDASI-BT assesses symptom severity and symptom interference [20]. The MDASI-BT reports a mean score of the 22 symptoms. Subscales of activity related interference, mood related interference, and symptom scores of affect, cognition, neurologic, treatment-related, GI, and generalized disease.

Emotional state was assessed with three Patient Reported Outcomes Measurement Information System (PROMIS®) measures. The PROMIS Short Form v1.0—Depression 8a assesses mood, views of self, and social cognition [21]. The PROMIS Short Form v1.0—Anxiety 8a reports fear, hyperarousal, and anxious misery [22]. The PROMIS Short Form v1.0—Psychosocial Impact-Positive 8a assesses the positive psychosocial outcomes of illness such as greater life appreciation, interpersonal relationships, and personal resources [23].

Additionally, an open response, Body Image Feedback Form (supplementary material) was provided to the participants. It allowed participants to respond to open-ended questions about their body image and cosmetic concerns, and the effects of the tumor and its treatment on their lives.

### Statistical analysis

IBM SPSS Version 25 was used for the statistical analysis [24]. Descriptive statistics were used for patient demographics and questionnaire scores. Independent t-tests, chi-square tests, and one way ANOVAs were used to compare groups. The relationship between body image and body image investment was evaluated using Pearson’s correlation coefficients. Stepwise multiple linear regression was used to examine which PROMIS measures are significantly associated with body image. Similarly, stepwise multiple linear regression was used to determine MDASI-BT 6 symptom subscales and 2 interference subscales best predict body image. Reliability of the BIS was also evaluated by calculating its Cronbach’s alpha. Significance was set at *p* < 0.05. Qualitative analysis was conducted using MAXQDA 2018 (VERBI Software, 2017) for text coding of response data on the Body Image Feedback form to identify recurring themes in body image concerns.

## Results

One hundred patients were enrolled and completed the BIS and ASI-R. Patients’ age ranged from 23 to 74 years old (median 48 years), and 56% of patients were male. The most common diagnosis was glioblastoma (32%), and 30% had low grade (WHO I–II) malignancies. The median time from diagnosis was 5 years (range 0–22), and 85% of the participants had a KPS ≥ 80. 66% of patients were overweight or obese, and all participants had undergone brain tumor surgery. Patient characteristics are presented in Table 1.

**Table 1:** Patient characteristics at the time of enrollment (N = 100)

Patient characteristics		
Age	Median (range)	48 (23–74)
		%
Sex	Male	56
	Female	44
KPS	≤ 70	6
	80	21
	≥ 90	64
	Missing	9
Grade	Grade I	4
	Grade II	26
	Grade III	35
	Grade IV	35
Diagnosis	Glioblastoma	32
	Anaplastic astrocytoma	20
	Anaplastic oligodendroglioma	9
	Ependymoma (WHO II–III)	8
	Meningioma (WHO I–III)	2
	Medulloblastoma	2
	Low grade glioma (WHO I–II)	21
	Rare^a^	6
BMI	Underweight	1
	Normal/healthy weight	30
	Overweight	35
	Obese	31
	Unknown	3
Receiving steroid medication	Yes	9
	No	84
	Unknown	7
Recurrent disease	Yes	37
	No	63
Prior therapy	Radiation	83
	Chemotherapy	78
Years from diagnosis	Median (range)	5.0 (0–22)

^a^Includes central neurocytoma, pineoblastoma, pleomorphic xanthoastrocytoma, anaplastic pleomorphic xanthoastrocytoma, anaplastic glioneuronal tumor, papillary glioneuronal tumor

The BIS score was used to evaluate body image dissatisfaction. The median BIS score was 5.0 (range 0–27) with a mean of 7.0 (SD = 6.7). Clinically significant body image dissatisfaction (BIS score ≥ 10) was present in 28% (95% CI 19–37%) with 14% of patients reporting no body image dissatisfaction (BIS = 0). The relationship between BIS, demographic, and clinical factors were assessed (Table 2). Age was significantly correlated with BIS scores (*r* = − 0.24, 95% CI − 0.44, − 0.05, *p* = 0.015), with younger patients having increased body image dissatisfaction. The BIS demonstrated very good reliability in the PBT population with a Cronbach’s alpha of 0.91 with all ten items contributing to its reliability.

**Table 2:** Univariate test results for relationships with BIS scores

Demographic		Mean BIS score	*p* value
Sex	Male	5.9	0.063
	Female	8.4	
Radiation	Yes	7.0	0.911
	No	7.2	
Chemotherapy	Yes	6.7	0.373
	No	8.1	
Recurrence	Yes	7.9	0.314
	No	6.5	
Tumor grade	I–II	7.6	0.488
	III	7.6	
	IV	5.9	
KPS	≤ 80	8.1	0.364
	90	6.0	
	100	7.8	
Treatment phase	New diagnosis	3.0	0.831
	On treatment	6.9	
	Follow-up	7.2	

Variable	Correlation with BIS	*p* value
Age at enrollment	*r* = − 0.24	0.015
BMI at enrollment	*r* = 0.195	0.056

The ASI-R evaluated body image investment. The ASI-R composite scores ranged from 1.5 to 4.7 (mean = median = 2.9 (SD = 0.6)). Subscale scores for Self-Evaluative salience ranged from 1.2 to 4.5 (mean = 2.6 (SD = 0.7), median = 2.5); and Motivational salience ranged from 1.9 to 5.0 (mean = 3.3 (SD = 0.7), median = 3.4). The BIS score was significantly correlated with the ASI-R Composite score (*r* = 0.53, 95% CI 0.37, 0.66), the ASI-R Self-Evaluative score (*r* = 0.65, 95% CI 0.51, 0.77), but not with the ASI-R Motivational score (*r* = 0.14, 95% CI − 0.06, 0.33). As body image dissatisfaction increased so did the negativity of the patient’s belief about their appearance. Cronbach’s alpha for the ASI-R composite, and self-evaluative and motivational subscales were 0.87, 0.85, and 0.81 respectively.

Table 3 shows the responses on the three PROMIS measures and the MDASI-BT categorized by those with no body image dissatisfaction against those with body image dissatisfaction. The mean PROMIS Depression score was 48.4 (SD = 8.9), the mean PROMIS Anxiety score was 49.4 (SD = 9.9), and the mean PROMIS Psychosocial Illness Impact Positive score was 48.9 (SD = 9.7).

**Table 3:** Average scores for BIS, the symptom, and emotional state measures

	Mean^a^	Standard deviation
BIS total score	7.03	6.799
PROMIS psychosocial t-score	48.91	9.662
PROMIS depression t-score	48.416	8.9430
PROMIS anxiety t-score	49.383	9.8882
MDASI-BT affective factor	2.226	2.2021
MDASI-BT cognitive factor	1.633	2.0183
MDASI-BT neurologic factor	1.072	1.5907
MDASI-BT treatment related factor	1.553	1.8572
MDASI-BT generalized disease factor	1.144	1.6734
MDASI-BT GI factor	0.596	1.5542
MDASI-BT activity-related interference	1.713	2.0909
MDASI-BT mood-related interference	1.656	2.0009

^a^N = 94 patients

Using stepwise multiple regression the PROMIS Psychosocial Illness Impact Positive and PROMIS Anxiety scores were the measures that were the most strongly related to BIS accounting for 33% of the variability in the BIS score  (Table 4). The PROMIS Psychosocial Impact Positive was negatively correlated with the BIS score, suggesting that an improved outlook on the psychosocial impact of their disease was associated with less body image dissatisfaction.

**Table 4:** Association between BIS score, symptom, and emotional state measures

Coefficients^a^					
Model	B^b^	SEB^c^	β^d^	t	*p* value
(Constant)	6.457	7.325		0.881	0.381
PROMIS psychosocial t-score	− 0.266	0.078	− 0.378	− 3.427	0.001
PROMIS depression t-score	0.082	0.114	0.108	0.718	0.475
PROMIS anxiety t-score	0.182	0.093	0.265	1.948	0.055
MDASI-BT affective factor	0.358	0.658	0.116	0.544	0.588
MDASI-BT cognitive factor	− 0.220	0.394	− 0.065	− 0.558	0.578
MDASI-BT neurologic factor	0.364	0.566	0.085	0.643	0.522
MDASI-BT treatment related factor	0.597	0.542	0.163	1.102	0.274
MDASI-BT generalized disease factor	− 0.097	0.627	− 0.024	− 0.154	0.878
MDASI-BT GI factor	− 1.082	0.524	− 0.247	− 2.064	0.042
MDASI-BT activity-related interference	0.538	0.509	0.166	1.058	0.293
MDASI-BT mood-related interference	− 0.773	0.691	− 0.228	− 1.119	0.266

^a^Dependent Variable: BIS total score

^b^B, unstandardized regression coefficient

^c^SEB, standard error of B

^d^β, standardized regression coefficient

Using the MDASI-BT subscales, stepwise multiple regression showed that only the MDASI-BT mood-related interference significantly predict BIS scores accounting for 19% of the BIS variability. It was positively correlated with BIS scores suggesting that more mood interference was associated with more negative body image.

### Qualitative analysis

The open-ended Body Image Feedback Form was completed by seventy-two patients and provided additional insight into the patients’ perspectives. Five main themes (Lifestyle changes, Symptom Effects, Changes in Appearance, Positive/Negative outlook) characterized patients body image challenges. Twenty-six patients (36%) contributed to the most prevalent theme relating to lifestyle factors (mobility issues (23/26;89%), daily routine impacted (4/26;15%), changes in relationships (3/26;12%), and loss of independence (1/26;4%). One patient reported, “It has affected my daily routine or just doing the things in life like walking, kneeling, steps, hills, things we take for granted (59 years old female)”. Patients described common short and long- term symptoms (weight gain (11/24; 46%), vision problems (5/24; 21%), pain (4/24; 17%), fatigue (2/24; 8%) impacting their quality of life. Additionally, treatment and surgery impacted changes in appearance (32%; n = 23), as patients mentioned visible symptoms (hair loss (16/23; 70%), indentation on the head (6/23; 26%), and dry skin (2/23; 9%). One patient reported, “Whenever I meet someone, I feel like they will judge me for my scar on my head and I do feel especially unattractive because of the scar in my head (36 years old male)”. Only 3 out of the 23 (13%) patients affirmed making a lifestyle adjustment to accept unavoidable changes to their appearance. A negative outlook [mood changes (5/9; 56%), self-conscious (4/9; 44%)], difficulty dealing with changes (2/9; 22%) endorsed by nine patients (13%) described their concerns with long-term physical changes, however a positive change in perspective (n = 8; 11%), acceptance of changes (5/8; 63%), exercise (2/8; 25%), hope/importance of physical health/enjoyment of life (1/13%) provided new meaning to patients’ outlook. On the open-ended questionnaires, fifteen patients (21%) reported no body image concerns.

## Discussion

This protocol investigates a previously unstudied QoL concern for patients with PBT [9]. Body image has been shown to be an important QoL mediator in patients with many types of cancer, affecting patients’ mood, social interactions, sexuality, and causing physical and psychological distress. Emerging evidence supports active intervention in these areas to improve patient outcomes [14].

The current data supports the importance of body image concerns in the PBT population as well, with 28% of patients having clinically significant body image dissatisfaction, and only 14% of patients noting none on the BIS measure. Age and BMI showed small mean differences in BIS scores, with younger patients or patients with a larger BMI reporting more body image dissatisfaction. No difference was seen in the body image outcomes in terms of sex, tumor grade, performance status, or previous therapy.

The prevalence and amount of body image dissatisfaction among PBT patients presents at a similar level to that seen in other solid tumor populations. Harrington et al. [8] reported a mean BIS score of 6.13 in prostate cancer patients in various stages of their illness trajectory, compared to PBT patients with a mean BIS score of 7.0 [8]. Patients with prostate cancer who had received androgen deprivation therapy (ADT) significantly differed from those who were ADT naïve (BIS score of 6.97 versus 4.27). Rates of clinically significant body image dissatisfaction (BIS ≥ 10) were 27% in patients treated with ADT, and 14% in those without. The rate of patients who described no body image dissatisfaction (BIS = 0) was 16% vs 39% for ADT vs ADT-naïve respectively. Factors affecting body image dissatisfaction in this prostate cancer population were ADT exposure and BMI, but not age. Therefore, the rates of body image dissatisfaction in the PBT population parallel those seen in men with prostate cancer who have received ADT, an intervention that significantly affects patient quality of life [25]. Another study found rates of 51% of men with testicular cancer having minor changes in body image, with 10% noting moderate to severe change [5]. There was also no association between body image dissatisfaction and age [5, 8]. In the PBT patient sample, there was a positive correlation with BMI, as well as a negative correlation with age. The latter finding may in part be due to the age ranges available for analysis, as both prostate and testicular cancers are naturally skewed toward particular age groups.

It was hypothesized that parallels could be drawn between the disease related dysfunction and treatment effects in the head and neck cancer and PBT populations [9]. However, in a cross-sectional study of head and neck cancer patients, their mean BIS score was 4.93 (SD = 6.21), lower than the score reported by PBT patients [12]. The head and neck cancer patients completed an additional body image survey where 75% of patients reported feeling concerned or embarrassed by bodily changes related to their diagnosis, despite their lower mean BIS score [12]. Factors associated with body image dissatisfaction among head and neck cancer patients were age and time since diagnosis, with younger patients having more body image dissatisfaction. BIS score did not differ based on age or cancer type.

Similarly, a sample of patients with oral cavity cancers were found to have a mean BIS score of 2.51 (SD = 3.14) [26]. Correlations were seen between BIS and depression and anxiety, with depression as the strongest predictor of body image outcomes. Investment in appearance (using the ASI-R) scores ranged from 1.8 to 4.25 with a mean of 2.93 (SD = 0.56), similar to the mean response in the PBT patient sample. However, unlike the PBT patient population, the correlation between ASI-R and BIS for oral cavity was not statistically significant [26].

In the general head and neck population study, 33% of patients endorsed behavioral changes including reassurance seeking, increased grooming or checking behaviors, or avoidance of grooming due to heightened concern with appearance which is more in keeping with the results of the ASI-R in PBT patients [12]. However, it is possible that general head and neck cancer population may have more similar appearance and dysfunction concerns as a PBT than a select group with oral cavity cancers.

An analysis of body image dissatisfaction among patients with advanced cancers of the breast, GI, GU, head and neck, gynecologic, hematologic and respiratory system demonstrated a 58% rate of clinically significant body image dissatisfaction (BIS ≥ 10), which significantly correlated with increased physical and psychological distress and depression [6]. This relationship was similarly found in the PBT patient sample in regard to anxiety. Mosher et al. (2013) found that nearly half of women with metastatic breast cancer reported distress about appearance concerns [27]. Hair loss, scars, weight gain, lymphedema, and hyperpigmentation of the nail beds were sources of frustration and embarrassment for patients. Unlike PBT patients, however, in patients with other cancers, body image dissatisfaction was associated with increasing symptom burden [6]. Patients with advanced cancer that scored positive for body image dissatisfaction were more likely to rank changes in their body as equally or more important than fatigue [6]. In the PBT patient sample, the impact of a positive psychological outlook demonstrated the strongest relationship with the BIS, which suggests that a patient’s outlook on their disease has a significant impact on how they view any resulting changes in their body. This is further supported by evidence in the breast cancer population that patients with more positive body image have higher self-efficacy and coping [28].

With increasing longevity and survival in a variety of brain tumors, the potential impact of body image will become a more significant patient survivorship concern. The importance of this survivorship issue in other tumor sites is well recognized even amongst patients with poor prognosis [4, 6, 29]. For example, body image in women with metastatic breast cancer remains highly influential even with a shortened life expectancy [4]. Outcomes such as hair loss have been shown to be important QoL mediators in other cancer populations [28–30].

Establishing a model of body image dissatisfaction among PBT patients is of clinical importance and may allow for intervention in this patient population, as has been done in other tumor types [14, 30]. These body image models have established intervention recommendations for body image concerns including education, prostheses, cosmetic rehabilitation, beauty treatments, strength training, and cognitive behavioral therapy [5, 14, 31].

The BIS and ASI-R demonstrated good internal consistency and reliability in the PBT population and are useful measures for future analysis. A BIS score of ≥ 10 has been suggested as a clinical cutoff for body image dissatisfaction. It has been used in a variety of cancer pathologies, and patients with advance disease, supporting its use in PBTs, however, the optimum cutoff for the PBT population remains an important area of future research [6, 8, 17, 18]. While there was significant correlation between BIS and ASI-R composite and self-evaluative scores, this was not true with the motivational score. One explanation may be that the ASI-R motivational score measures potential effect. In essence, individuals who attend to their appearance are more likely to show correlation between measures of body image satisfaction or dissatisfaction and predictor variables. However, those individuals who do not attend to their appearance (i.e. have low ASI-R motivational scores), may be more likely to have no correlation, regardless of the measure, as body image does not impact their daily life. This may be an important differentiation to study in the future as the motivation and self-assessment capacity of PBT may differ from other cancer populations.

This study explored the prevalence of body image concerns in the previously unstudied PBT population. In determining the factors associated with body image concerns limitations of this study include patients numbers, single institution, and cross sectional design. Future research may focus on change in body image dissatisfaction over time to determine opportune time points for intervention, optimal cutoff values of body image scales, and primary CNS tumors of the spinal cord [32–34].

## Conclusion

Despite the potential significant impact of tumor and treatment effects on body image and subsequently self-esteem, sexuality, and interpersonal relationships in the PBT population, very little was known. The present study demonstrated that 28% of PBT patients are affected by clinically significant body image dissatisfaction. Exploratory analysis of related factors included BMI, age, anxiety, and patient outlook on the impact of their illness. Based on the knowledge of the significant QoL impact of body image in other tumor types, this data supports future research into the characteristics and interventions for this QoL issue among PBT patients.

## Electronic supplementary material

Below is the link to the electronic supplementary material.

Supplementary file1 (DOCX 15 kb)
