# Supplementary material for: The prevalence of altered body image in patients with primary brain tumors: an understudied population
Source: J Neurooncol. 2020 Feb 24;147(2):397–404. doi: 10.1007/s11060-020-03433-8 (PMC7136178; doi:10.1007/s11060-020-03433-8)
Supplement: Supplementary file 1 — Supplementary file1 (DOCX 15 kb) [file 11060_2020_3433_MOESM1_ESM.docx]

FEEDBACK FORM:

Which changes in your appearance have you noticed since your diagnosis? (Check all that apply)

| - Hair loss | - Weight gain | - Change in symmetry of your face |
| --- | --- | --- |
| - Eyebrow loss | - Weight loss | - Swelling of your face |
| - Change in skin | - Visible scar | - Change in walking |
| - Bruising | - Change in the symmetry of your eyes | - Change in the use of your arms or legs |
| - Other: | | |

How have the changes above affected you? (Fill in the box below)
